# Supplementary material for: IRimage: open source software for processing images from infrared thermal cameras
Source: PeerJ Comput Sci. 2022 May 10;8:e977. doi: 10.7717/peerj-cs.977 (PMC9138121; doi:10.7717/peerj-cs.977)
Supplement: Supplemental Information 1 — Images were all those found in Wikimedia Commons taken with FLIR cameras and available as unmodified JPG files. Images were downloaded on 16 Jun 2019 from https://commons.wikimedia.org/wiki/Category:Photos_taken_with_FLIR_Systems. [file peerj-cs-08-977-s001.pdf]

Table S1. List of images used for the comparison between IRimage and FLIR Tools. Images were all those found in Wikimedia Commons taken with FLIR cameras and available as unmodified JPG files. Images were downloaded on 16 Jun 2019 from
https://commons.wikimedia.org/wiki/Category:Photos_taken_with_FLIR_Systems

#	Image name	Camera model	Author	License
1	200 deg neutral	ThermaCAM SC640	Basicinfrared	CC0
2	IRWaterCooler	InfraCAM Wester	Eclipse sx	CC-BY-3.0
3	Thermal image of four ducks swimming	FLIR i7	Zaereth	CC0
4	AFCIs Infrared	FLIR C2	Tenbergen	CC-BY-SA-4.0
5	Aqua Tower thermal image	Flir b60	Russ Miller- Johnson	CC0
6	Aqua Tower thermal imaging	Flir b60	Jim D'Aloisio	CC0
7	BillHotFlashThermography	ThermaCAM EX320	Hotflashhome	CC-BY-SA-3.0
8	Image thermique de l'émission d'un radiateur à travers un mur	FLIR E30bx	Hugues Crepin	CC-BY-SA-3.0
9	Infrared image of people in the laboratory	FLIR i60	Tomasz Kawalec	CC-BY-SA-4.0
10	IR Fussbodenheizung	ThermaCAM SC640	Herbertweidner	CC0
11	IR moving car	FLIR E60	Herbertweidner	CC0
12	IR moving mercedes	FLIR E60	Herbertweidner	CC0
13	IRWater	FLIR E60	Eclipse sx	CC-BY-SA-3.0
14	Kujawy wiatrak	FLIR T440	Tadmark5364	CC-BY-4.0
15	Linear load	InfraCAM Wester	Basicinfrared	CC0
16	Man in water - IR image	FLIR P640	Krzysztof Jakucy	CC-BY-SA-3.0
17	Solar halo thermal	FLIR T420	Разрывные	public domain
18	Steam Train Valves Thermal Image	FLIR T420	Christopher A.S. Harvey	CC-BY-SA-4.0
19	Termografía	InfraCAM Wester	Gemmaburt	CC-BY-SA-4.0
20	Thermal image of a group of grey-headed flying foxes during an extreme temperature event	FLIR E60bx	Justin Welbergen	CC-BY-SA-3.0
21	Thermal image of a juvenile grey-headed flying fox during an extreme temperature event	FLIR E60bx	Justin Welbergen	CC-BY-SA-3.0
22	Thermogramme infiltrométrie	FLIR B360	DanFernbanck	CC-BY-SA-4.0
23	Thermographie de rue	FLIR E30bx	Hugues Crepin	CC-BY-SA-3.0
24	Thermographie photovoltaique	FLIR E30bx	Hugues Crepin	CC-BY-SA-3.0
25	Videocamera Termica	FLIR E40	Carla Vacchi	CC-BY-SA-4.0
26	Windmill Thermal Image	FLIR E60	Black Hills Thermal Imaging	CC-BY-SA-3.0
