# Supplementary material for: IRimage: open source software for processing images from infrared thermal cameras
Source: PeerJ Comput Sci. 2022 May 10;8:e977. doi: 10.7717/peerj-cs.977 (PMC9138121; doi:10.7717/peerj-cs.977)
Supplement: Supplemental Information 4 — Scatter plots comparing the temperature values of all the pixels in each image obtained using IRimage vs. the reference temperature values obtained from the original images using the manufacturer’s software FLIR Tools. Data is shown for 25 out of the 26 tested images; comparison was not possible for image #4 because it could not be opened with FLIR Tools. Information about the images used is available in Table S2. [file peerj-cs-08-977-s004.pdf]

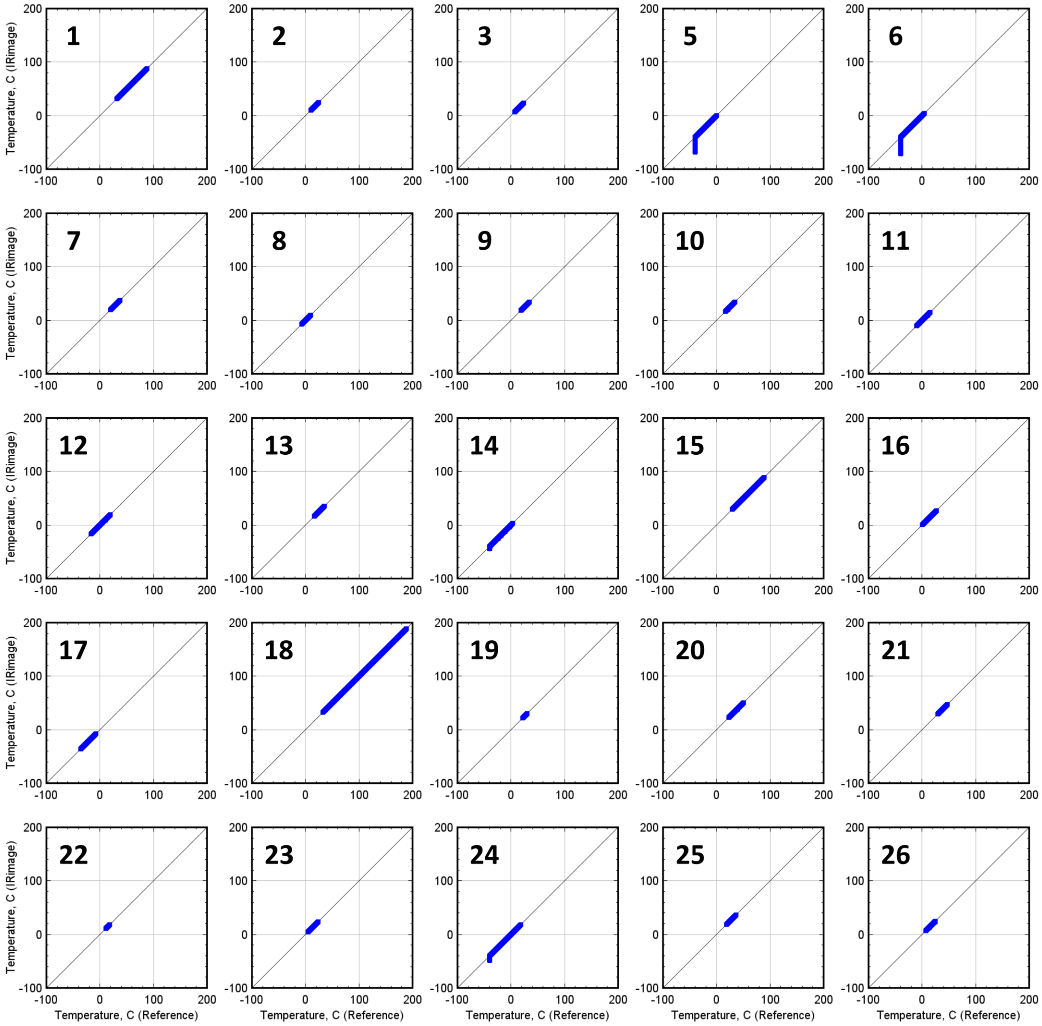

Figure S1. Results of the comparison between IRimage and FLIR Tools. Scatter plots comparing the temperature values of all the pixels in each image obtained using IRimage *vs.* the reference temperature values obtained from the original images using the manufacturer's software FLIR Tools. Data is shown for 25 out of the 26 tested images; comparison was not possible for image #4 because it could not be opened with FLIR Tools. Information about the images used is available in Table S2.
