# Supplementary material for: IRimage: open source software for processing images from infrared thermal cameras
Source: PeerJ Comput Sci. 2022 May 10;8:e977. doi: 10.7717/peerj-cs.977 (PMC9138121; doi:10.7717/peerj-cs.977)
Supplement: Supplemental Information 5 — Normally, the values of user-defined parameters are set in the camera before the images are captured, and these values are either kept fixed or are updated manually as conditions change. IRimage allows the user to set these parameters individually for each image, which can be necessary under fluctuating conditions. This Figure compares the results processing a set of images using either fixed parameters (i.e., using mean values for air temperature and relative humidity and reflected temperature) or variable parameters (i.e., using values measured or estimated for each image, as shown in Fig. 5 in the main text). (A-B) Absolute air temperature (grey solid line), absolute leaf temperature obtained with fixed parameters (dotted red/blue lines), and absolute leaf temperature obtained with variable parameters (solid red/blue lines), in the well-watered (A) and water-stressed (B) plants. (C-D) Leaf-to-air temperature difference (DeltaT) obtained with fixed parameters (dotted red/blue lines), and DeltaT obtained with variable parameters (solid red/blue lines), in the well-watered (C) and water-stressed (D) plants. The gray shaded area indicates the night. While temperature values obtained with these two methods seem quite similar in absolute values (A-B), DeltaT curves reveal the effect of parameter selection on the resulting values (C-D): when air temperature and humidity within the greenhouse were close to the mean values, both methods returned similar results, but using fixed parameters yielded leaf temperatures values almost 1° C higher or lower when environmental conditions deviated from the average values. [file peerj-cs-08-977-s005.pdf]

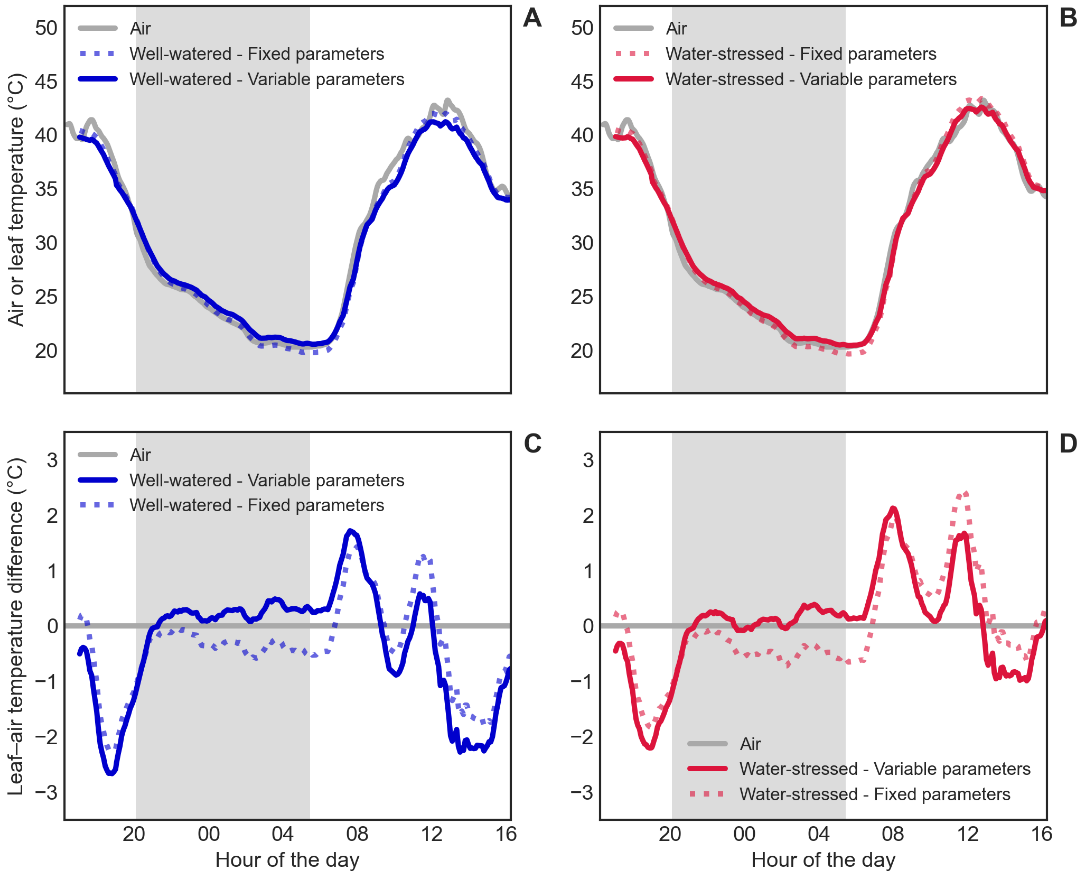

Figure S2. Comparison of leaf temperature values obtained with either fixed parameters or variable parameters. Normally, the values of user-defined parameters are set in the camera before the images are captured, and these values are either kept fixed or are updated manually as conditions change. IRimage allows the user to set these parameters individually for each image, which can be necessary under fluctuating conditions. This Figure compares the results processing a set of images using either fixed parameters (*i.e.*, using mean values for air temperature and relative humidity and reflected temperature) or variable parameters (*i.e.*, using values measured or estimated for each image, as shown in Fig. 5 in the main text). (A-B) Absolute air temperature (grey solid line), absolute leaf temperature obtained with fixed parameters (dotted red/blue lines), and absolute leaf temperature obtained with variable parameters (solid red/blue lines), in the well-watered (A) and water-stressed (B) plants. (C-D) Leaf-to-air temperature difference (ΔT) obtained with fixed parameters (dotted red/blue lines), and ΔT obtained with variable parameters (solid red/blue lines), in the well-watered (C) and water-stressed (D) plants. The gray shaded area indicates the night. While temperature values obtained with these two methods seem quite similar in absolute values (A-B), ΔT curves reveal the effect of parameter selection on the resulting values (C-D): when air temperature and humidity within the greenhouse were close to the mean values, both methods returned similar results, but using fixed parameters yielded leaf temperatures values almost 1°C higher or lower when environmental conditions deviated from the average values.
